# Impact of ECG gating methods on the assessment of left ventricular cardiac function using PET/MRI

**DOI:** 10.1007/s12350-022-03177-0

**Published:** 2022-12-29

**Authors:** Sazan Rasul, Raffaella Calabretta

**Affiliations:** Department of Biomedical Imaging and Image-Guided Therapy, Division of Nuclear Medicine, Medical University of Vienna, Waehringer Guertel 18-20, 1090 Vienna, Austria

Left ventricle (LV) cardiac function is correlated to cardiac end-diastolic and end-systolic volumes (EDV, ESV) as well to global LV function, expressed as LV ejection fraction (LVEF). These parameters have a significant influence on cardiac morbidity and mortality, and thus, their thorough assessment has a considerable prognostic value in patients with heart diseases.^1^ In particular, the estimation of LV cardiac function in individuals having coronary artery disease (CAD) yields important information about the disease burden. Therefore, it plays a critical role in the subsequent treatment and clinical management of patients, and has the greatest predictive value for the disease outcome.^2^ Furthermore, modifications of LV size and functional parameters are related to occurrence of heart failure, which is characterized by a LV remodeling.^3,4^ Accurate assessment of LV functional parameters requires the analysis of cardiac cycle and, in particular, of its phases: systole, which includes iso-volumetric contraction and ejection, and diastole, with active and passive relaxation.^5,6^ Currently, several noninvasive routinely used imaging modalities such as cardiac magnetic resonance imaging (CMR), echocardiography, positron emission tomography (PET), single photon emission computed tomography (SPECT), and computer tomography (CT) allow the assessment of LV cardiac function and motion during the cardiac cycle through electrocardiogram (ECG)-gated acquisitions.^7^ Among them, the CMR is considered the gold standard, because of its high temporal and spatial resolution.^8^ In the nuclear medicine practice, combined scanner such as PET/MRI performs a simultaneous execution of both imaging techniques, PET and MRI. Indeed, modern PET scanners with cardiac ECG gating imaging and list-mode data acquisition allow the precise definition of cardiac phases for cardiac function assessment, facilitate proper cross-validation analyses between CMR and PET scanners, and are fundamental for executing cardiac cine MR.^9^

The cross-validation analyses between various available ECG-gated techniques might avoid dependence on a particular device, when an examination with a certain machine is not possible due to its availability, patient-related reasons, or technical issues.^10,11^ In this context, combined PET/MRI allows simultaneous estimation of LV function with PET and CMR and offers an ideal direct comparison of values obtained from both methods.^12^

In this issue of the *Journal of Nuclear Cardiology*, Villagran Asiares et al^13^ investigated the impact of three ECG gating methods on the assessment of LV cardiac functional parameters such as EDV, ESV, and LVEF, in an enhanced cross-validation multimodal study. The authors analyzed 30 patients (97% male) with known coronary chronic total occlusion, previous to revascularization procedure, underwent PET/MRI with 2-deoxy-2-[^18^F]fluoro-d-glucose (2-[^18^F]FDG), in order to evaluate myocardial viability. The acquisition imaging protocol was the same for all patients. Conventional multi-slice 2-dimensional short axis CINE MRI-sequences were performed in order to acquire the reference values of LV functional parameters. Subsequently, an ECG gated with 8 phases, list-mode PET scan was conducted 60 minutes after the intravenous injection of 330 ± 32 MBq of 2-[^18^F]FDG. The ECG signal was recorded with MR-compatible 3-lead electrodes and was used for both, CMR acquisition and ECG-gated PET reconstructions. Considering that the presence of arrhythmias, which are not uncommon in patients with coronary artery disease, and the detection of irregular and aberrant R waves that utterly interfere with cardiac phases and may affect the estimation of LV function, different ECG gating methods were used to improve this comparative analysis. Regarding cardiac ECG-gated PET imaging, the authors precisely analyzed three ECG gating methods with 8 phases: standard gating (STD) with fixed number of gates per beat, beat rejection gating (STD-BR), which was like STD but rejected all abnormal R–R intervals caused by arrhythmias or artefacts with an acceptance window manually defined on the R–R intervals distribution for each patient, and fixed width gating (FW) that preserved the duration of each gate along the whole acquisition.^13^ The authors chose these ECG gating methods because STD and STD-BR are already available in PET/MRI system and the FW assumes that the systolic phase remains physiologically constant between different cardiac cycles.

Results of their correlation analysis generally showed a high correlation between CMR- and PET-enhanced EDS, ESV, and LVEF values acquired from a hybrid PET/MR system. In particular, this analysis demonstrated that the better correlation with CMR was for ESVs obtained by PET compared with PET EDVs. Regarding the ECG gating, the STD-BR method showed the best correlation, while the STD was the weakest. In addition, the agreement analysis underlined that the PET-system in all three ECG gating methods underestimated all LV functional parameters measured. In more detail, all ECG methods underestimated significantly the EDVs, whereas STD and FW underestimated markedly the LVEF. Nevertheless, based on Bland–Altman plots, the authors performed the intra-individual agreement between these two imaging modalities with all three ECG-guided methods showing broad relative limits of agreement (up to 70%) with no clear differences between the STD-BR and FW methods, whereas the STD method displayed the strongest disagreement.^13^

This study examines the impact of different PET cardiac ECG gating methods on LV cardiac function assessment using PET/MRI imaging in patients with CAD. The authors should be congratulated for conducting this interesting analysis and for selecting a topic of great clinical and diagnostic relevance in the setting of cardiac imaging. They demonstrated for the first time the relevance of the width of the beat acceptance window, not only in PET images reconstruction, but also in the acquisition of cardiac CINE MRI-sequences, affecting the accuracy of parameters analyzed.

One of the greatest strengths of this work is that the authors have expanded and linked their analyses to previous studies published in this field and concluded the interchangeability of cardiac function measurements between different imaging modalities, such as PET/MR, PET/CT, PET, and MR, is not feasible. Indeed, in a multimodal cross-validation, the authors presented numerous previously published comparative studies supporting the outcome of their study regarding the high correlations in measurement of LV cardiac functional parameters with various imaging techniques^12,14–18^ and the presence of several biases was similar to the study by Li et al^11^. Nevertheless, in contrast to these studies, this work focuses more on the impact of the several ECG gating approaches on the assessment of cardiac function by PET, using CMR-based assessment as a reference.

Moreover, the paper thoroughly illustrates the most relevant reasons for the discrepancies between PET- and CMR-derived cardiac volumes and LVEF values in the combined PET/MRI system. Among them, Villagran Asiares et al^13^ explained that PET and CMR are two different techniques with distinct geometric models of the heart. As a consequence, different responses to changes in heart rate and subsequently a lower number of effective gates may lead to a smoothing effect on PET images, resulting in an underestimation of LV cardiac function values extracted from PET compared with parameters gained from CMR. Furthermore, intra-scan fluctuations in heart rate due to the acquisition timing mismatch between PET and CMR and the utilization of CINE sequences with two- and not three-dimensional scans to provide the reference values of LV cardiac function have been discussed by the authors as major causes contributing significantly to the discrepancies between PET and CMR measurements of cardiac function values.

Another issue limiting the outcomes of this study and might be responsible for the discrepancies between PET and CMR assessment is the employment of only 8-phase ECG-based cardiac gating, as this method results in smaller values and, in particular, lower EF of the PET scanner than the use of 16 or 32 phases, as illustrated by Germano et al^19^. Furthermore, the reader-dependent manual delineation of the LV in both PET and CMR scanner with consequent inter-observer variability is a concern that could further limit the study findings, as outlined by the authors.

Essentially, this study highlighted the valuable contribution of various ECG gating methods and the need for rigorous ECG quality control when evaluating LV cardiac function using PET/MRI. Initiatively, some replacements to ECG-triggered cardiac gating, such as utilization of acoustic- or seismocardiography-based cardiac triggering, have already been presented in other researches.^20,21^

Despite these limitations, the current study of Villagran Asiares et al represents a very important step toward incrementing diagnostic role of different ECG gating methods in the setting of cardiac imaging. LV cardiac function parameters determined with a combined PET/MRI system had high correlations but underestimated values as well as wide limits of agreement, depending on the ECG gating methods used in both techniques, PET and CMR. Since the most significant differences were found in ECG gating method, which are prone to highly irregular beats, the authors caution against interchanging PET and CMR parameters for clinical purposes and recommend placing more emphasis on thorough quality control of ECG gating in daily clinical routines.

These considerations point to the importance of ECG gating in cardiac imaging and suggest that further studies on this topic are needed to enhance the diagnostic accuracy of functional LV imaging to appropriately stratify risk and improve disease outcomes.
